# Oxidative Stress in Alzheimer's Disease: Why Did Antioxidant Therapy Fail?

**DOI:** 10.1155/2014/427318

**Published:** 2014-01-05

**Authors:** Torbjörn Persson, Bogdan O. Popescu, Angel Cedazo-Minguez

**Affiliations:** ^1^KI-Alzheimer's Disease Research Center, Department of Neurobiology, Care Sciences and Society, Karolinska Institutet, 14186 Huddinge, Sweden; ^2^Department of Neurology, Colentina Clinical Hospital (CDPC), School of Medicine, “Carol Davila” University of Medicine and Pharmacy, 19-21 Sos. Stefan cel Mare, District 2, 020125 Bucharest, Romania; ^3^Laboratory of Molecular Medicine and Neuroscience, “Victor Babeş” National Institute of Pathology, 99-101 Splaiul Independenţei, District 5, 050096 Bucharest, Romania

## Abstract

Alzheimer's disease (AD) is the most common form of dementia in the elderly, with increasing prevalence and no disease-modifying treatment available yet. A remarkable amount of data supports the hypothesis that oxidative stress is an early and important pathogenic operator in AD. However, all clinical studies conducted to date did not prove a clear beneficial effect of antioxidant treatment in AD patients. In the current work, we review the current knowledge about oxidative stress in AD pathogeny and we suggest future paths that are worth to be explored in animal models and clinical studies, in order to get a better approach of oxidative imbalance in this inexorable neurodegenerative disease.

## 1. Introduction

Alzheimer's disease (AD) is the most common form of dementia and was carried by an estimated 35.6 million people in 2010 [1]. The number is expected to increase to about 115 million sufferers in year 2050. The challenges in AD research today include discovering methods to diagnose patients in an earlier stage and to find new treatments to prevent or cure the disease. There are some inherited forms of the AD, also known as Familial Alzheimer's Disease (FAD), caused by mutations in one of these three genes: Amyloid Precursor Protein (APP), Presenilin-1, and Presenilin-2. These mutations are all linked to the overproduction of amyloidogenic forms of the amyloid-*β* (A*β*), a peptide that is generated by a sequential cleavage of APP by the *β*- and *γ*-secretases [2]. The majority of AD cases (more than 95%) are, however, sporadic and cannot be explained by deterministic mutations. It is hypothesised that sporadic AD results from a combination between environmental factors and risk genes. The most important risk gene is apolipoprotein E (ApoE), encoding an important molecule in lipid metabolism. ApoE has in humans three different isoforms, *ε*2, *ε*3, and *ε*4. The *ε*4 allele is associated with increased risk for AD, while *ε*2 is considered to be protective. People with one copy of the *ε*4 allele have approximately three times higher risk of getting the disease, while homozygotes have 12 times higher risk [3]. Symptoms of AD are characterized by progressive decline in cognitive abilities such as memory, mood, and behaviour, which leads to social and mental disability. The underlying pathophysiology of AD includes loss of neurons and synapses in the cerebral cortex and parts of the subcortical areas [4]. Apoptosis is thought to be one of the mechanisms that lead to cell death in AD [5, 6]. In addition to this, AD brains also harbour extracellular amyloid depositions containing A*β*, so-called plaques, and intracellular neurofibrillary tangles containing hyperphosphorylated tau protein [7, 8]. Other hallmarks associated with AD pathology are neuroinflammation [9] with activation of microglia and higher levels of inflammatory proteins in the brain and oxidative stress, which is thought to be one of the earliest events in the disease pathogenesis [10]. In this review, we summarise the evidences linking oxidative stress to AD and the possibilities of using antioxidant therapies for treatment.

## 2. Oxidative Stress

Oxidative stress is defined as the imbalance between the generation of reactive oxygen/nitrogen species (ROS/RNS) and the cells ability to neutralize them by the antioxidant defence. The main source of ROS is in the electron transport chain (ETC) at the mitochondrial inner membrane where energy is generated in the form of ATP. During ETC, the electrons are transferred from NADH and FADH_2_, via four membrane bound complexes (complex I–IV), to oxygen, which finally generates water [11]. As a natural event, some electrons are leaking from the inner membrane and react with oxygen to form superoxide anions (O_2_^∙−^). This can further react to generate other forms of ROS such as hydrogen peroxide (H_2_O_2_), hydroxyl radicals (OH^∙^), and hydroxyl ions (OH^−^). The RNS is generated when O_2_^∙−^ reacts with nitric oxide (NO) to form peroxynitrite (ONOO^−^). These can then in consecutive reactions form other types of RNS, like nitrogen dioxide (^∙^NO_2_) and nitrosoperoxycarbonate (ONOOCO_2_^−^).

Other sources of ROS and RNS in brain are astrocytes and microglia that produce these species when activated and also in reactions catalyzed by redox active metal ions such as copper and iron. The ROS/RNS are capable of damaging and modifying several types of macromolecules within the cell, including DNA, RNA, lipids, and proteins. These modifications can then generate even more potent reactive molecules [12].

The impact of DNA oxidation could be detrimental since it can affect transcription and replication of important genes [13]. One of the major markers for DNA oxidation is 8-hydroxydeoxyguanosine (8-OH-dG) [14] that is generated by oxidation of the nucleoside guanosine by OH^∙^. Oxidation to RNA occurs in a similar way with oxidation of RNA bases. The most prevalent one is the RNA analogue to 8-OH-dG and 8-hydroxyguanosine (8-OHG) [15]. The RNA is considered to be more susceptible to oxidation due to its cellular location with a higher proximity to the ROS generation. The major consequence of the RNA oxidation is probably breaking of the nucleotide strand, but it can also cause ribosomal dysfunction [16].

Oxidation of lipids could be deleterious since it has the potential of damaging the cell membranes. The unsaturated fatty acids in the membranes are the most susceptible to oxidation and become peroxidized when attacked by OH^∙^. Isoprostanes are formed when polyunsaturated fatty acids (PUFAs) get peroxidized [17] and their levels are considered to be a very accurate measure of oxidative stress [18]. Lipid oxidations also form reactive aldehydes such as malondialdehyde (MDA) and 4-hydroxynonenal (HNE). These can get attached to proteins and thereby disrupt their function [12]. Oxidation of proteins can yield several different outcomes such as side-chain oxidation, backbone fragmentation, unfolding and misfolding, and loss of activity [19]. All amino acids are susceptible to oxidation, but they are more or less sensitive. Cysteines and methionines are easily oxidized; however, many of these oxidations are reversible by disulfide reductases. There are examples of irreversible modifications *in vivo* such as S-carboxymethylcysteine (CMC) and S-(2-Succinyl)cysteine (2-SC) [20, 21]. These modifications are fumarate and dicarbonyl groups, respectively, that are covalently bound to cysteine residues. Oxidation of lysine, proline, and arginine threonine renders in carbonyl derivatives that are used as markers of ROS-mediated protein oxidation [22].

As mentioned, the mitochondria are the main source of ROS and thus also a vulnerable target to oxidative damage. Impairment of the mitochondrial membranes and proteins can generate even more ROS that could cause damage to the mitochondrial DNA and lead to cell death by apoptosis [23]. An important sensor for the initiation of apoptosis by oxidative stress is ASK-1. Its activity is mainly regulated by the oxidoreductase Thioredoxin-1 (Trx-1), a highly redox sensitive protein that binds ASK-1 in a reduced state [24]. When Trx-1 is oxidized, the binding to ASK-1 is disrupted leading to activation of the downstream c-Jun N-terminal Kinase (JNK) pathway of apoptosis [25]. The activity of ASK-1 can also be modulated by other redox proteins including Glutaredoxin (Grx), heat-shock proteins, and glutathione S-transferase (GST). Another regulator of oxidative-mediated apoptosis is p53, which translocates to the nucleus to activate proapoptotic genes during oxidative stress [26].

## 3. Oxidative Stress in Alzheimer's Disease

A major sign of aging is oxidative stress and a significant amount of evidence has shown that oxidative stress is an important pathogenic factor in AD. A study of brains in different disease stages showed increased levels of 4-hydroxyhexenal (HHE), a marker of lipid peroxidation, already in the early stages of the disease [27]. This was also seen for other markers, such as F_2_-isoprostane and F_4_-neuroprostane, when comparing levels in frontal, parietal, and occipital lobes between controls, individuals with mild cognitive impairment (MCI), and late AD patients [28]. When comparing levels of F_2_-isoprostanes in frontal poles from AD brains with brains from Parkinson's disease (PD) patients and schizophrenia patients (SCHI), no differences were seen between PD, SCHI, and controls, while the levels were increased in AD [29]. Peroxidation of lipids can create electrophilic aldehydes that are highly reactive and easily modify proteins. Acrolein is an example of such an aldehydic lipid peroxidation product that is increased in AD brains [30] and can create damage to mitochondria by inhibiting respiration and modulating tau phosphorylation [31]. Similar observations were made for oxidations of DNA and RNA when measuring levels of 8-hydroxyguanine (8-OHG) in hippocampus and superior and middle temporal gyri [32]. The oxidation of DNA in AD brains is also true for mitochondrial DNA [33] which is more sensitive to oxidation than the nuclear DNA [34].

Markers of protein oxidation, such as protein carbonyls, were increased in AD brains in areas with established histopathological AD features [35]. Protein carbonyls and 3-nitrotyrosine (3-NT) levels were increased in frontal cortex of individuals with MCI, mild AD, and AD, with no difference among the disease stages, a finding which supports the idea of oxidative stress as an early event in the AD [36].

The markers for oxidative stress in brain are also reflected in the cerebrospinal fluid (CSF), where modified macromolecules can be detected. The levels of F_2_-isoprostanes were increased in CSF from patients with probable AD compared to controls. Interestingly, the increase was not found in patients with Amyotrophic Lateral Sclerosis, which is another neurodegenerative disorder with oxidative stress involvement [37]. The DNA oxidation marker 8-OH-dG was also significantly increased compared to controls in CSF removed from the lateral ventricle at autopsy [38]. Oxidation and nitration of proteins were measured in CSF from AD patients and controls [39] and the concentration of 3-NT was found increased in AD and was negatively correlated with MMSE (minimental state examination) score. Focus has also been given to find markers of oxidative stress in serum or plasma, in order to efficiently diagnose patients at risk or in an AD earlier stage. However, in this research line, the results are so far inconclusive. One study showed that both malondialdehyde (MDA) and protein carbonyls were increased in serum from a group of patients with either MCI or diagnosed AD, in comparison to healthy controls. This study did not differentiate between MCI and AD patients [40]. Another study showed higher serum levels of F_2_-isoprostanes, compared to age-matched controls, while protein carbonyls were unchanged [41]. However, when F_2_-isoprostanes were measured in plasma of patients with AD, MCI, and PD and in healthy controls, no significant difference was found [42]. Interestingly, in a study on rats, no correlation was found between F_2_-isoprostane levels in brain and plasma, suggesting that markers in blood do not reflect the oxidative status in the brain [43]. Most studies on DNA markers of oxidative stress in blood have been done in lymphocytes and leukocytes. Studies have found increased levels in peripheral lymphocytes from AD patients [44] and also in leukocytes from both MCI and AD patients [45]. The RNA oxidation marker 8-OHG has also been measured in plasma, but the levels were not altered between AD and control subjects. These levels did not correlate with the levels in CSF either [46].

With the accumulating evidence for oxidative stress as an important pathogenic factor in AD, theories have emerged speculating that oxidative stress is involved in the initiation of the disease. In fact, oxidative damage was found to be the first observable event in the disease progression among all AD hallmarks [10]. This has also been seen in transgenic animal models, where oxidative stress markers appear before A*β* accumulation [47]. As oxidative stress is a part of normal aging, a difference between AD and normal aging could be caused by a different ability of the neurons to deal with increased ROS production. The brain is particularly vulnerable to oxidative damage and has high oxygen consumption. About 20% of all oxygen and 25% of all glucose are consumed by the cerebral functions [48]. The brain also contains a relatively high content of polyunsaturated fatty acids (PUFA) that are more sensitive to oxidation [49]. On the other hand, levels of the antioxidant defence in the brain are modest, the fact which renders neurons especially sensitive to a disturbance in the balance between antioxidants and production of ROS. Moreover, the brain also has a high content of redox active metals, which can promote the formation of ROS and has been linked to AD pathology.

There is a clear relationship between A*β* and oxidative stress. It is known that A*β* can cause increased production of ROS and also damage mitochondria, which can lead to further enhanced production. These effects can be seen in the brain of the triple-transgenic mouse model of AD, wherein lipid peroxidation is increased and simultaneously GSH and Vitamin E levels are decreased [47]. Interestingly, this change was observed before any plaque pathology. In another mouse model, expressing double mutant of APP, induction of oxidative stress and inflammation by thiamine deficiency aggravated the plaque pathology and increased the levels of A*β*(1-42) [50]. In both AD patients and transgenic mice, A*β* was shown to interact with A*β*-binding alcohol dehydrogenase (ABAD) in the mitochondria [51]. This interaction caused increased ROS formation, mitochondrial dysfunction, and ultimately apoptosis [52].

In fact, apoptosis is considered to be the major type of cell death in AD and there are many other mechanistic links between oxidative stress and apoptosis reported in AD models. A study on fibroblasts from AD patients and control subjects found that two antiapoptotic proteins (HSP 60 and Vimentin) were oxidized in response to treatment with A*β* peptide. Similarly, we observed in neuroblastoma cells how TRX-1 and GRX-1, two antiapoptotic protein previously mentioned, get oxidized when cells were treated with A*β*(1-42) [53]. A*β* treatment also leads to lower levels of glutathione (GSH) and increased levels of markers for apoptosis. In addition, more HNE bound to the proapoptotic protein p53 was found in the inferior parietal lobule AD patients compared to controls [54], a modification that can trigger apoptosis [55].

Oxidative stress has been also linked to the genetic risk factor ApoE4, both in AD patients and in healthy subjects. In hippocampus of AD patients, ApoE4 was associated with higher oxidative stress and reduced antioxidant enzyme activity [56]. In healthy individuals, higher levels of isoprostanes in CSF were associated with the *ε*4 genotype [57]. In fact, *in vitro* evidence has shown that the different isoforms of ApoE possess different antioxidant properties, with *ε*2 being the most and *ε*4 the least protective, in response to A*β* treatment [58].

## 4. Antioxidants in Alzheimer's Disease 

Not only are several markers of oxidative stress increased in AD but also there is also evidence for lower antioxidant power in the brain, CSF, and blood. The most prevalent antioxidant in most brain cells is GSH. It can react with ROS and oxidized products forming glutathione disulphide (GSSG), either catalysed by Glutathione Peroxidase (GPx) or independently. The GSSG can then be converted back to reduced GSH by Glutathione Reductase (GR). Studies of human brains have indicated that the ratio between reduced and oxidized glutathione (GSH/GSSG) is decreased in affected brain regions of AD patients compared to controls [59]. This was also observed in lymphocytes from AD patients [60] and in hippocampus from patients with MCI [61]. However, a recent study that measured GSH levels in anterior and posterior cingulate in MCI and control patients using proton magnetic resonance spectroscopy, found higher levels in MCI patients compared to controls [62]. Higher GSH levels were also associated with poorer performance in cognitive tests. This could be a compensatory mechanism, where more GSH are produced in response to the increased oxidation. In line with these data, the levels, but not activities, of GR were elevated in hippocampus from patients with amnestic MCI compared to controls [61]. The same study also measured GPx but no significant differences were detected. When different antioxidant enzymes were analyzed in plasma, GPx activity in patients with early stage dementia was lower compared to controls [63]. This observation can be detected already in plasma or serum from MCI patients [64, 65]. However, when the activity of GPx was measured in erythrocytes from MCI and AD patients, there was no change in MCI and an increase in AD, compared to controls. The same study found also decreased GR activities in samples from both MCI and AD individuals, compared to controls. In order to better appraise the effect on the GSH cycle, the ratio between GR and GPx activity was calculated. This study showed that the ratio was decreased in MCI and further decreased in AD, suggesting a progressive reduction of the capacity to recycle GSH among the disease. In a population-based study with a follow-up 4 years later, a significant correlation was found between activity of GPx in red blood cells and cognitive decline [66]. However, regarding GPx in AD brain, the results have been inconsistent [67].

One of the most important endogenous antioxidants, *α*-tocopherol (Vitamin E), is lipophilic and is thought to be protective against lipid peroxidation [68]. It was found to be decreased in plasma from mild AD patients compared to controls [69] which also has been observed for patients with MCI [70]. A study done on CSF reported a reduction in *α*-tocopherol levels in AD [71]. However, another study analysing postmortem ventricular CSF from patients with and without dementia showed no difference in levels between the groups but found a small but significant correlation between *α*-tocopherol levels and performance in perceptual speed test and negative correlation with neuritic plaques. High Vitamin E levels in plasma have also been associated with reduced risk of AD in advanced age [72]. Another vitamin, ascorbic acid (Vitamin C), is necessary for the reactivation of Vitamin E. Ascorbic acid is one of the most important water-soluble antioxidants and levels in plasma that were reported to be reduced in both MCI and AD patients, compared to controls [64]. A decrease in nutritional antioxidants is presumably not due to malnutrition but rather reflects the increased oxidative stress apparent in the periphery, which leads to higher consumption of antioxidants. A cross-sectional study in 74 mildly demented and 158 age-matched controls found an association between dementia and low levels of Vitamin C in plasma. However, no association was found with Vitamin E levels [73].

Another important micronutrient for the antioxidant defence is selenium (Se). Selenium is an essential trace element that is a part of the amino acid selenocysteine and functions as a cofactor in important antioxidant enzymes such as (GPx) and Thioredoxin Reductase (TrxR). In a 9-year longitudinal study in patients between 60 and 71 years of age, cognitive decline was found to be associated with a decrease in plasma Se over time [74]. The plasma levels were also significantly lower when comparing diagnosed AD patients with healthy control subjects. Yet, studies have demonstrated that plasma levels of Se are independent of brain levels [75]. Nevertheless, analysis of Se levels in autopsy brain tissue of AD patients has not yet given a consistent result [67]. The levels and activity of the seleno-containing enzyme, TrxR, which catalyzes the reduction of Trx-1, have been shown to be increased in AD brains [60, 76]. On the other hand, we reported that in AD Trx-1 is decreased in neurons but upregulated in glial cells [53]. In the same study we observed that AD brains showed reduced neuronal levels of Grx-1, an enzyme with similar function as Trx1. Recently we demonstrated that an 80 amino acid long cleavage product of Trx1, called Thioredoxin 80 (Trx80), was drastically reduced in AD brains [77]. This decrease could also be detected in CSF and was observable already in patients with MCI. The analysis of CSF samples could discriminate patients with stable MCI from MCI patients that later progressed to AD. When Trx1 is cleaved to Trx80 it looses its reductive capacity [78] but functions as an inhibitor of A*β* polymerization [77].

Superoxide dismutase is part of the initial defence against ROS and catalyses the conversion of O_2_^∙−^ to H_2_O_2_ and oxygen (O_2_). The activity of SOD in serum was reduced in both MCI and AD patients compared to controls and was negatively correlated to the lipid peroxidation marker MDA [65]. The reduction in SOD activity was also reported in hippocampus from MCI patients; however, total levels of SOD were reduced as well [61]. The H_2_O_2_ generated by SOD can be converted to water and oxygen by catalase. In patients with early stage dementia, catalase activity was decreased in plasma with no change in protein levels [63]. Nevertheless, in erythrocytes, the activity of catalase was increased in AD compared to controls. No such change was detected in MCI [79].

The incidence of AD is higher in women than in men and a suggested reason has been the estrogen deficiency in postmenopausal women [80]. Low estrogen levels in CSF have also been been associated with higher A*β* levels in brain of female AD patients [81]. Estrogen has antioxidant properties and we and others have shown that it can protect neurons against oxidative stress induced damaged caused by both A*β* and ApoE4 [82, 83].

## 5. Antioxidants Therapies in Alzheimer's Disease

With the accumulative evidence for oxidative stress as an important factor in AD, several trials have been conducted with the aim of treating or preventing the disease using antioxidants. A summary of clinical trials can be found in Table 1.

All epidemiological studies that have shown a correlation between low nutritional status and AD have led to prevention studies and clinical trials with some of these essential compounds. Studies on the effect of vitamins have been ambiguous. In a one-year follow-up study, AD patients taking cholinesterase inhibitors as treatment were supplemented with Vitamin C (1000 mg/day) and Vitamin E (400 IU/day). The supplementation decreased lipid peroxidation markers in CSF; however, no difference was observed in cognition when using the minimental state examination (MMSE) [84]. In another study, patients with mild to moderate AD were treated for 16 weeks with a mix of Vitamin E (800 IU/day), Vitamin C (500 mg/day), and *α*-lipoic acid (900 mg/day) [85]. Here, CSF levels of lipid peroxidation were also reduced, but there were no effect on A*β*42 or p-tau levels. Surprisingly, the patients receiving the antioxidant mixture declined faster in MMSE score than the placebo group.

Selenium has in clinical trials most often been tested in combination with other compounds. In one small study, 15 geriatric patients were receiving inorganic Se (8 mg), organic Se (45 *μ*g), and Vitamin E (400) or placebo. The patients receiving the treatment formulation improved in the several cognitive parameters using the Sandoz Clinical Assessment Geriatric Scale [86]. Of note, however, is that the dosage of selenium was very high. A larger study with selenium and Vitamin E using selenomethionine (200 *μ*g) is now being conducted in the PREADVISE trial. It includes more than 10,000 men between 60 and 90 years old with no neurological or psychiatric illness and will study the effect of the treatment on preventing AD and other brain disorders [87].

The natural polyphenolic compound curcumin, derived from the plant *Curcuma* Long Lin, is reported to have antioxidant properties and to inhibit A*β* aggregation [88, 89]. It has shown effect on oxidative stress and plaque burden in transgenic mice and also improved performance in cognitive tests [90, 91]. Due to these findings, the compound has been tested in clinical trials. Disappointingly, the trials have not yet yielded a positive outcome. In a randomized controlled study, patients diagnosed with probable or possible AD were given 1 or 4 g curcumin per day orally [92]. The result showed no changes in MMSE score, serum levels of A*β*(1-40), or F_2_-isoprostane levels in plasma. The study was rather short (6 months) and biomarkers were only measured in blood, which are not necessarily correlated with the levels in the brain or CSF, as mentioned above. In another study, patients with mild to moderate AD were receiving a mixture of curcuminoids orally, 2 or 4 g, per day [93]. The study was longer than the previous one and also measured biomarkers in CSF. However, no significant differences were found in neither neuropsychological test nor in the biomarker measurements. However, the plasma levels of curcumin after drug administration were low suggesting a poor uptake from the gastrointestinal tract. Curcumin has also been tested in healthy subjects [94]. A small group of middle-aged people were given a small dose (80 mg/day) of lipidated curcumin orally for four weeks to test for various health beneficial outcomes. This treatment induced an increase in saliva antioxidant status. In plasma, catalase activity was also increased but no effect on GPx or SOD activities were found. Importantly, the treatment significantly decreased A*β*(1-40) in plasma. The conductors of the study predicted a better uptake with lipidated curcumin compared to nonlipidated form. However, the plasma levels of curcumin were not tested in this study.

Another natural compound that has been tried for prevention of memory decline is extracted from the leaves of the Ginkgo biloba tree. It has been used in Chinese traditional medicine and has several reported properties, including antioxidant activity [95]. In a large randomized controlled trial, with a median follow-up time of 6.1 years, 3069 elderly persons above 72 years of age were given 120 mg of Ginkgo biloba standardized extract (EGb 761) twice daily. The main outcome measures were rates of change over time in various neuropsychological tests. The results did not show any difference in cognitive decline between treatment and control group [96]. A similar trial studied the effect on the rate of progression to AD after long-term use of EGb 761 or placebo in 2854 elderly subjects, whom spontaneously had reported memory complains [97]. This study did not find any significant positive effect of EGb 761.

Approaches have been made with nutraceutical formulations containing several active compounds, such as Memory XL. It contains the vitamins folic acid, Vitamin B12, and Vitamin E; acetyl-l-carnitine, a compound that maintains mitochondria function and has antioxidant properties, and S-adenosylmethionine (SAM) and N-acetyl cysteine (NAC), two compounds involved in the generation of the endogenous antioxidant GSH. In one study, this formulation or placebo was given to individuals between 18 and 86 with no signs of dementia [98]. Outcome measurements were changed in performance on California Verbal Learning Test II and Trail-Making Test. After 3 months, participants receiving the formulation had improved both clinically and significantly in the test scores. Interestingly, no effect was seen in participants above 74 years of age. In this study report, the authors suggest that this could be due to nutritional deficiencies, low absorption of the active ingredients, or an age-related cognitive decline in these subjects. As mentioned already, mitochondria are particularly vulnerable to oxidative damage. Therefore, strategies have been tested to target mitochondria using compounds with antioxidant properties. Coenzyme Q10 (CoQ10) is a quinone located in the mitochondria and is involved in the electron transport chain. CoQ10 prevented cognitive impairment in a rat model of sporadic AD generated by intracerebroventricular injection of streptozotocin [99]. However, CoQ10 possesses a poor ability to pass into the brain via the blood-brain barrier [100]. A synthetic analogue of CoQ10, Idebenone, which easier passes the blood-brain barrier, has been used in clinical trials. In a multicenter study, 563 patients with mild AD were treated with different doses of idebenone or placebo for one year. However, the treatment did not show any changes in cognitive decline compared to the placebo group [101]. In order to have a proper effect of CoQ10 or analogues thereof, the mitochondria have to retain an intact electron transport chain. To overcome this problem, a compound called MitoQ has been developed. It passes the blood-brain barrier, accumulates well in the mitochondria, and it has been shown to operate without an intact electron transport chain [102]. The compound has been tested in a transgenic mouse model of AD where it prevented cognitive decline and AD-related pathological changes in the brain. So far, it has not been tested in clinical trials for AD. Hormone replacement therapy with estrogen has been used in clinical trials as treatment of AD in women. In a randomized, placebo controlled study, 120 women with mild to moderate AD were treated with estrogen for one year [103]. However, the treatment neither slowed down the disease progression nor improved any of the global, cognitive, or functional outcomes.

Proline-rich polypeptides, derived from colostrum (Colostrinin), were first described as stimulators of the immune response [104]. Later, in PC12 cells, it was also shown to decrease the generation of ROS caused by the lipid peroxidation product 4-hydroxynonenal and to prevent activation of JNK and p53, two important regulators of apoptosis [105]. Recently, Colostrinin was also demonstrated *in vitro* to increase the activities of GPx and GR while having no effect on lipid peroxidation, suggesting that Colostrinin could modulate the endogenous early defence mechanisms rather than preventing oxidative damage [106]. Colostrinin has been used in clinical trials for treatment of AD. In one study, thirty-three patients with mild to moderately severe AD were given 100 *μ*g Colostrinin every other day for three weeks, followed by a two-week break. This was repeated for 16–28 months. No placebo group was included. In the study, the only outcome measurement was the MMSE score. The results showed a small but significant stabilization of MMSE score with Colostrinin treatment with only mild side effects [107]. In a larger trial, 105 patients with mild to moderate AD were treated for 15 weeks, with Colostrinin or placebo, followed by an open-label phase for 15 weeks [108]. In this study, the treatment with Colostrinin demonstrated as well a stabilizing effect on the cognitive functions in AD Assessment Scale-cognitive portion (ADAS-Cog) score and on functions of daily living in a test for Instrumental Activities of Daily Living (IADL). The major effect was seen in patients with mild AD, supporting the notion that early intervention would be the most favourable. These findings are promising, but additional studies are necessary to explain the mode of action in the treated patients.

## 6. Discussion

Clearly, oxidative stress is a significant element in AD pathogenesis. In addition, several indications suggest that it is one of the earliest signs of the disease. Possibly, lack of protection against ROS production in the aging brain could be one triggering cause of AD and a driving force in disease progression. Altered A*β* production is the consequence of the mutation in FAD, which is mimicked in most transgenic mice models used in AD research. These animals have an early increased oxidative stress in the brain, implying that oxidative stress is one of the first consequences of A*β* overproduction in the brain. On the other hand, experiments have shown that oxidative stress can cause both increased production and accumulation of A*β* [109–111], which also supports the inverse relationship. It is alluring to speculate that oxidative stress in a key feature in this process and perhaps a driving force in the sporadic forms of the disease, accelerating the spread of pathology. The pressure from oxidative stress in the aging brain in combination with a lowered antioxidant defence creates a harmful combination that could disturb functions and damage organelles such as mitochondria. This would eventually lead to loss of synapses and cell death that give rise to the clinical symptoms associated with AD. For these reasons, therapies using antioxidants still hold great promise. However, results so far have been rather disappointing and most studies have failed due to various reasons.

Regarding trace elements and vitamins, an important factor is the dosage, considering that antioxidant compounds can act as prooxidants under deleterious conditions [112]. Supplementation of one antioxidant in too big amounts might therefore be harmful. Many antioxidants are also highly dependent on other antioxidants, such as Vitamin C or GSH, in order for them to be active. This is a potential reason for which administration of one, single, nutritional antioxidant has not worked as a therapeutic strategy for AD. Another reason for which clinical trials have not yet yielded a definite positive result could possibly be that the interventions are started to late in disease progress. Since oxidative stress starts very early in the disease progression, it is likely that treatment will be most effective if started early as well, before other damaging processes take over in a “point of no return.” In addition, it is important to remember that the brain is separated from the periphery by the blood-brain barrier, which prevents molecules from leaving or entering the central nervous system, a fact that must be considered when studying disease mechanisms as well as conducting clinical trials. What is observed in the periphery regarding oxidative stress markers and antioxidants might not be reflected in the brain or the CSF. When surveying studies on antioxidant levels in blood, it is clear that results will differ depending on the applied analysis (for plasma, serum, or blood cells). This is an issue that must be considered when clinical trials are conducted and antioxidant status is used as an outcome measurement. Antioxidants in blood could be potentially used for clinical diagnosis as well, the fact which makes a proper evaluation of the true blood antioxidant status utterly important.

Since oxidative stress is part of normal aging, a key question is to define whether interventions towards boosting the general antioxidant capacity of the brain should be used as general preventive approaches against neurodegeneration. This question is similar for other neuroprotective strategies, such as growth factor enhancement. However, such strategies are potent enough to stop progressive neurodegenerative disorders as AD is questionable. This is probably true for all the treatments targeting a single feature of the disease. Since AD is such a heterogeneous disorder, multimodal or combinatory strategies (including antioxidant therapy) should be explored.

## Figures and Tables

**Table 1:** Summary of clinical trials with antioxidants to prevent or treat MCI and AD.

Compounds	Subject population	Intervention	References
Vitamin C and Vitamin E	23 patients with probable AD stably taking cholinesterase inhibitors.	Daily supplementation; Vitamin C (1000 mg) and Vitamin E (400 IU) for one year.	[84]

Vitamin C, Vitamin E, *α*-lipoic acid, CoQ10	78 subjects with mild to moderate AD	Vitamin C (500 mg/day), Vitamin E (800 IU/day), *α*-lipoic acid (900 mg/day) or CoQ10 (400 mg, 3 times/day) or placebo for 16 weeks.	[85]

Selenium and Vitamin E	30 geriatric patients	Administration of sodium selenate (8 mg) daily in two doses, organic selenium (45 *μ*g/day) and Vitamin E (400 mg) in two daily doses, or placebo for 1 year	[86]

Selenium or Vitamin E	10.000 male subjects between 60 and 90 years old with no neurological or psychiatric illness	(Ongoing study, NCT00040378) Daily doses of 400 IU Vitamin E or 200 *μ*g selenomethionine or placebo.	[87]

Curcumin	34 patients with probable or possible AD	Patients received 1 or 4 g of curcumin or placebo, orally once daily for 6 months.	[92]

Curcumin	36 subjects with mild to moderate AD.	Administration of curcumin C3 complex, 2 or 4 g/day for 48 weeks. A control group were receiving placebo for 24 weeks followed by either 2 or 4 g curcumin per day for another 24 weeks.	[93]

Curcumin	38 healthy middle-aged people of 40–60 years old.	Lipidated curcumin (80 mg/day) or placebo were given for 4 weeks.	[94]

Ginko biloba	3069 elderly people above 72 years without dementia symptoms.	Two daily doses of G.biloba extract EGb 761 (120 mg) or placebo with a median follow-up time of 6.1 years.	[96]

Ginko biloba	2854 subjects, 70 years or older, with spontaneously reported memory complaints to primary care physician.	Two daily doses of G.biloba extract EGb 761 (120 mg) or placebo with a follow-up time of 5 years.	[97]

Memory XL (folic acid, Vitamin B12, Vitamin E, acetyl-L-carnitine, SAM, NAC)	115 participants of both genders, 18–86 years with no signs of dementia or clinical memory difficulties	One daily dose of two Memory XL tablets containing folic acid (400 *μ*g), Vitamin B12 (6 *μ*g), Vitamin E (30 IU), SAM (400 mg), NAC (600 mg), and acetyl-L-carnitine (500 mg) for 2 weeks or 3 months followed by an open-label phase for 2 weeks or 3 months.	[98]

Ebenone	536 subjects above 50 years with diagnosed probable AD.	One-year treatment with daily doses of 120, 240, or 360 mg Idebenone or placebo.	[101]

Estrogen	120 women diagnosed with probable AD. All women had hysterectomies performed.	A single daily dose of 0.625 or 1.25 mg of Premarin (conjugated equine estrogens) or placebo were given for 1 year followed by a 3-month single blind placebo washout phase.	[103]

Colostrinin	33 patients with mild to moderately severe AD.	Colostrinin treatment containing 100 *μ*g PRP complex every other day for 3 weeks followed by a 2-week break. This was repeated for 16–24 months.	[107]

Colostrinin	105 patients with mild to moderate AD.	Treatment with 100 *μ*g Colostrinin or placebo for three weeks followed by a 2-week break. This was repeated for 15 weeks followed by a 15-week open-label phase.	[108]
